# Supplementary figures and images for: High density optical neuroimaging predicts surgeons’s subjective experience and skill levels
Source: PLoS One. 2021 Feb 18;16(2):e0247117. doi: 10.1371/journal.pone.0247117 (PMC7891714; doi:10.1371/journal.pone.0247117)

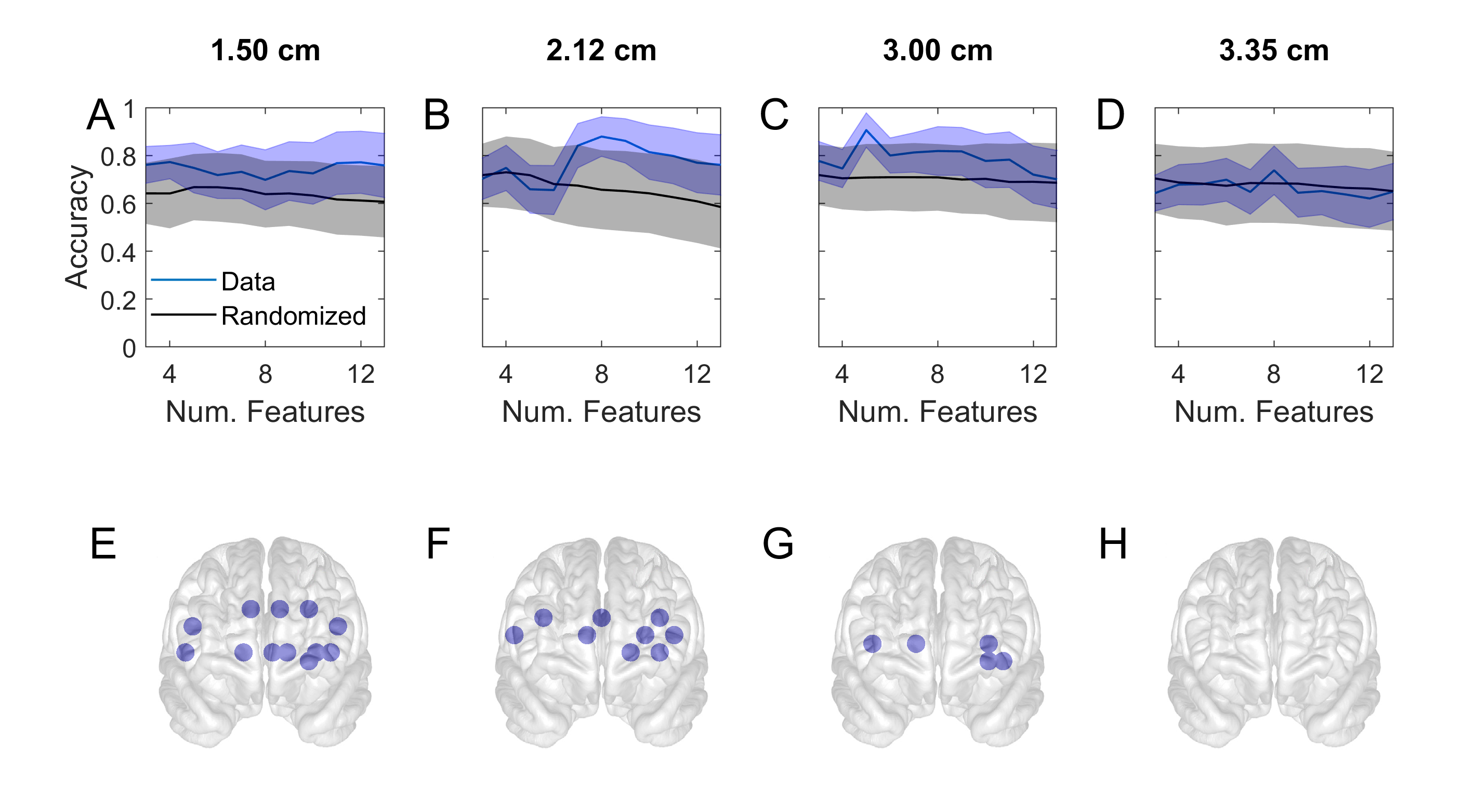

Supplement: S1 Fig — This figure shows the same results as in Fig 5 but it is for attending subjects. No channels locations are indicated in H since the accuracy remained at chance level. (TIFF) [file pone.0247117.s001.tiff]
