# Supplementary material for: High density optical neuroimaging predicts surgeons’s subjective experience and skill levels
Source: PLoS One. 2021 Feb 18;16(2):e0247117. doi: 10.1371/journal.pone.0247117 (PMC7891714; doi:10.1371/journal.pone.0247117)
Supplement: S1 Table — (DOCX) [file pone.0247117.s002.docx]

| **S1 Table:Subject Demographics, Task Completion Times and Task NASA-TLX Scores** | | | | | | | | | |
| --- | --- | --- | --- | --- | --- | --- | --- | --- | --- |
| **Subject #** | **Group** | **SE In Number  of Years** | **LSE  In Number** | **Task 1  Completion* (Seconds)** | **Task 2  Completion* (Seconds)** | **Pre-Test NASA-TLX Sum** | **Task 1 NASA-TLX Sum** | **Task 2 NASA-TLX Sum** |  |
| 1 | Undergraduate Student | 0 | 0 | 326 | 279 | 97 | 94 | 93 |  |
| 2 | Undergraduate Student | 0 | 0 | 315 | 137 | 74 | 61 | 79 |  |
| 3 | Undergraduate Student | 0 | 0 | 300 | Failed | 70 | 55 | 70 |  |
| 4 | Undergraduate Student | 0 | 0 | Failed | 312 | 53 | 87 | 54 |  |
| 5 | Undergraduate Student | 0 | 0 | 360 | Failed | 81 | 67 | 97 |  |
| 6 | Undergraduate Student | 0 | 0 | Failed | Failed | 75 | 72 | 78 |  |
| 7 | Undergraduate Student | 0 | 0 | 278 | 150 | 82 | 88 | 61 |  |
| 8 | Undergraduate Student | 0 | 0 | 242 | 201 | 72 | 41 | 44 |  |
| 9 | Undergraduate Student | 0 | 0 | 247 | 122 | 69 | 68 | 55 |  |
| 10 | Undergraduate Student | 0 | 0 | 341 | 158 | 58 | 73 | 46 |  |
| 11 | Undergraduate Student | 0 | 0 | Failed | Failed | 95 | 77 | 65 |  |
| 12 | Undergraduate Student | 0 | 0 | 268 | 229 | - | 66 | 61 |  |
| 13 | Undergraduate Student | 0 | 0 | 163 | 181 | 57 | 31 | 52 |  |
| 14 | Undergraduate Student | 0 | 0 | 207 | 124 | 72 | 67 | 61 |  |
| 15 | Undergraduate Student | 0 | 0 | 303 | 253 | 79 | 73 | 94 |  |
| 16 | Undergraduate Student | 0 | 0 | 156 | 187 | 41 | 30 | 33 |  |
| 17 | UndergraduateStudent | 0 | 0 | Failed | 218 | 100 | 109 | 96 |  |
| 18 | Surgery Resident | 0.25 | 0 | 232 | 359 | 69 | 65 | 85 |  |
| 19 | Surgery Resident | 1.5 | 0 | Failed | 255 | 69 | 92 | 81 |  |
| 20 | Surgery Resident | 3 | 0 | 294 | 220 | 90 | 74 | 61 |  |
| 21 | Surgery Resident | 1.5 | 5 | 278 | 88 | 59 | 55 | 48 |  |
| 22 | Attending Physician | 9 | 8 | Failed | 140 | - | 40 | 49 |  |
| 23 | Attending Physician | 6 | 30 | 192 | 345 | - | 77 | 103 |  |
| 24 | Attending Physician | 5 | 30 | 220 | 145 | - | 56 | 46 |  |
| 25 | Surgery Resident | 2.75 | 40 | Failed | 326 | 108 | 65 | 73 |  |
| 26 | Attending Physician | 6 | 45 | 269 | 170 | - | 51 | 51 |  |
| 27 | Attending Physician | 7 | 75 | 160 | 83 | 49 | 61 | 30 |  |
| 28 | Attending Physician | 12 | 75 | 172 | Unattended | - | 30 | Unattended |  |
| 29 | Attending Physician | 30 | 100 | 313 | 268 | - | 44 | 54 |  |
| 30 | Attending Physician | 13 | 150 | 151 | 100 | - | 21 | 18 |  |
| 31 | Attending Physician | 13 | 250 | 200 | 294 | - | 35 | 84 |  |
| 32 | Attending Physician | 15 | 350 | 154 | 120 | - | 17 | 28 |  |
| 33 | Attending Physician | 16 | 350 | 250 | 127 | - | 17 | 12 |  |
| SE:Surgery Experience, LSE:Laparoscopic Surgery Experience *The tasks that were not completed in 6 minutes, were marked as failed. The tasks that were preferentially unattended by some of the subjects were marked as unattended | | | | | | | | | |
