# Supplementary material for: High density optical neuroimaging predicts surgeons’s subjective experience and skill levels
Source: PLoS One. 2021 Feb 18;16(2):e0247117. doi: 10.1371/journal.pone.0247117 (PMC7891714; doi:10.1371/journal.pone.0247117)
Supplement: Attachment — Submitted filename: PLOS ONE Review Comments.pdf [file pone.0247117.s006.pdf]

PLOS ONE Review Comments

Summary

In this study, the authors used fNIRS to measure the induced cognitive load on prefrontal cortex of expert and novice surgeons. The authors examined the potential predictive power of these fNIRS measurements for determining the cognitive load as well as the expertise of their participants in performing two laparoscopic surgery tasks: peg transfer and threading.

Major Comments:

Data Analysis:

How did the authors compute the hemoglobin concentrations (e.g., beer-lambert, etc.)? Also, the preprocessing of NIRS time series appear to solely include bandpass filtering without any baseline normalization and detrending (the latter for prevent potential non-stationarity in time series). Another issue is with regards to the use of standard deviation (3 in their case) for artefacts attenuation. This step is quite unconventional and is not (to the best of reviewer's knowledge) practiced in the literature. The authors are encouraged to consult [1] for a comprehensive review of NIRS preprocessing.

[1] Tak, S. and Ye, J.C., 2014. Statistical analysis of fNIRS data: a comprehensive review. *Neuroimage*, *85*, pp.72-91.

In addition, the authors also appear to use their measured NIRS standard deviation (i.e., after they preprocessed it) for quantification of the brain activation. This is also very unconventional. Specifically, what the authors refer to as "PFC activation" throughout the manuscript is indeed the deviation of the channels' activation from the average (observed/induced) PFC activity. In fact, such deviation could still be present without any sufficient/significant induced PFC activation by the task. The authors are strongly encouraged to consult [2,3] for measures used for NIRS quantification.

[2] Naseer, N. and Hong, K.S., 2015. fNIRS-based brain-computer interfaces: a review. *Frontiers in human neuroscience*, *9*, p.3.

[3] Keshmiri, S., Sumioka, H., Yamazaki, R. and Ishiguro, H., 2018. Differential entropy preserves variational information of near-infrared spectroscopy time series associated with working memory. *Frontiers in neuroinformatics*, *12*, p.33.

With regard to the use of short-distance channels "to perform superficial signal regression (SSR)" on long-distance channels, it is not clear what methodology/approach the authors adapted in their study. Some of the available approaches are:

[4] Fekete, T., Rubin, D., Carlson, J.M. and Mujica-Parodi, L.R., 2011. The NIRS analysis package: noise reduction and statistical inference. *PloS one*, *6*(9), p.e24322.

[5] Zhang, Y., Brooks, D.H., Franceschini, M.A. and Boas, D.A., 2005. Eigenvector-based spatial filtering for reduction of physiological interference in diffuse optical imaging. *Journal of biomedical optics*, *10*(1), p.011014.

[6] Kohno, S., Miyai, I., Seiyama, A., Oda, I., Ishikawa, A., Tsuneishi, S., Amita, T. and Shimizu, K., 2007. Removal of the skin blood flow artifact in functional near-infrared spectroscopic imaging data through independent component analysis. *Journal of biomedical optics*, *12*(6), p.062111.

[7] Haeussinger, F.B., Dresler, T., Heinzel, S., Schecklmann, M., Fallgatter, A.J. and Ehlis, A.C., 2014. Reconstructing functional near-infrared spectroscopy (fNIRS) signals impaired by extra-cranial confounds: an easy-to-use filter method. *NeuroImage*, *95*, pp.69-79.

[8] Gagnon, L., Perdue, K., Greve, D.N., Goldenholz, D., Kaskhedikar, G. and Boas, D.A., 2011. Improved recovery of the hemodynamic response in diffuse optical imaging using short optode separations and state-space modeling. *Neuroimage*, *56*(3), pp.1362-1371.

[9] Keshmiri, S., Sumioka, H., Okubo, M. and Ishiguro, H., 2019. An Information-Theoretic Approach to Quantitative Analysis of the Correspondence Between Skin Blood Flow and Functional Near-Infrared Spectroscopy Measurement in Prefrontal Cortex Activity. *Frontiers in neuroscience*, *13*, p.79.

Analysis Steps with Machine Learning:

Although the authors mentioned the use of signal's standard deviation for quantifying the brain activation, they then switched to its mean for ML-based classification (page 8: "For this purpose feature matrices were built with each row (an observation) representing the mean of the prefrontal activation over an episode"). Such inconsistencies and mixing of measures/metrics make quite difficult to realize the potential underlying property of the signal based on which the results have been derived.

The authors also mentioned that (page 9) "The feature types were prioritised by using the Pearson correlation between the observations and the labels, a standard feature-selection technique" – Do authors refer to the channels as features? If so, this step actually decided on which subset of channels to be used as inputs to their ML model.

The authors continued by explaining that (page 9) "We then chose a small group of features from the prioritised list and used it to train Support Vector Machines (SVM) with linear kernels." – How did the authors decide this "small group of features?" Did they apply such utilities as "features importance" that are available through ML libraries? If so, what criterion/criteria was/were used to determine the level of significance of feature scores? (while using the term "feature," I am assuming "selected channels" as per authors' earlier explanation).

The authors also used 5-fold cross-validation for testing the accuracy of their model. As the authors explained, every participant in their study participated in two different tasks (i.e., Peg Transfer and

Threading). As such, did the authors ensure that data from the same individual were not present in both train and test sets while applying 5-fold cross-validation? This is an important issue while performing such analyses since data from the same individuals should not be expected to be highly different between the two tasks which could, in turn, results in overestimation of the model's accuracy.

Result:

As one of their hypotheses, the authors stated (page 9) "that there would be differences in the activations due to the different sampling depths of the channels with different separation distances." – The effect of channel separation and skin- other than cortical-blood-flow is a well-studied subject. In fact, short-distance channels are not expected to represent cortical activity. Similarly, channels with distances larger than 3.5 cm have been also generally accepted to not produce reliable results due to the absorption of optical signals as it penetrates deeper to cortical tissues. Please consult [5-9] above for more in-depth results and discussion on this matter.

The authors' statement (page 10) "Figure 2 shows that in the Student subjects who had experienced higher task load also had higher PFC activations." that is quite repeated throughout the manuscript is not really valid since the authors used the standard deviation of the signal. In other words, these quantities are how individuals' PFC activity deviated from the averaged observed/induced PFC activation in their study.

With regard to results' presentation, the authors sufficed to such statements as (e.g., page 10) "The difference between high and low load subjects were statistically significant in both Tasks in the case of the shortest (1.5 cm, A and E) and the normal separation (3 cm, C and G) channels." while referring to the figures 2 through 7 without providing any descriptive statistics for their results. Precisely, it is not clear how the authors determined these results "were statistically significant?" What type of tests did they apply? What were the p-values, test-statistics, mean, standard deviation, confidence intervals, and effect-sizes associated with these tests? Did the authors corrected their p-values while determining their significance (e.g., Bonferroni, FDR, etc.)?

The authors stated that (page 11) "In addition, for the student subjects there was a pronounced asymmetry in the case of the deepest sampling channel (D and H), the activation on the left being significantly higher than on the right." – The reviewer encourages the authors to consult the studies related to the effect of short/long-distance channels on NIRS measurements that are listed above. In particular, the "deepest sampling channel" in the present study could fall within the range that is considered not suitable for studying the cortical activation.

With regard to the source localization presented in Figure 5, the authors used such statements as (page 11) "there is a hint of high activation localized near the top left …" – Such assertion are not justified unless the authors provide statistical evidence for the possibility of such activations that differ from the other regions.

Above shortcomings with regard to the results' representation also apply to the case of the results pertinent to ML-based results.

Discussion:

The authors stated that (page 13) "This difference, visible in most channel separations, was statistically significant in the 1.5 cm and most of the 3 cm separated channels (Figure 2)." – The authors did not present sufficient statistics for this claim (only presenting figures).

They also stated that (page 13) "in skilled subjects in the correlation of PFC activation with subjective task" however, the reviewer could not find/see these correlation analyses.

Another issue is with regard to hemispheric differences that authors referred to (page 14) "We found that response was greater in the left PFC of students (Figure 4D and H), ..." – Unless the authors perform statistical tests, such claims are not truly founded.

Other Comments:

The language of the manuscript requires a thorough auditing and proofread as it is not easy to follow and comprehend the study.

The quality of figures are very low and must be improved.

Please also break your Results Section into different subsections (e.g., one for test of significant differences, another for ML-based results, etc.) to help reader better follow and understand the results.
