# Supplementary material for: High density optical neuroimaging predicts surgeons’s subjective experience and skill levels
Source: PLoS One. 2021 Feb 18;16(2):e0247117. doi: 10.1371/journal.pone.0247117 (PMC7891714; doi:10.1371/journal.pone.0247117)
Supplement: Attachment — Submitted filename: Response to Reviewers.docx [file pone.0247117.s007.docx]

**We are grateful to reviewers for reading of our manuscript and their incisive and helpful comments. We agree with their comments and we introduced changes which, on our opinion, address all criticisms. As a result we believe that the new version of the manuscript is greatly improved.**

**Below we give the reviewer comments in bold. After each comments our response is described in detail in indented text.**

**Reviewer #1: The paper addresses an interesting topic with clinical
application. The flow of writing is good and easy to follow. The
statistical and machine learning approach are rigorous.

Recommendations: on the machine learning section, it is recommenced to
add more details on the feature selection. It is recommended to increase
the resolution of the figures.**

OUR RESPONSE: We have new details on feature selection in the new version. The new figures have high resolution.

**Reviewer #2: PLOS ONE Review Comments

Summary

In this study, the authors used fNIRS to measure the induced cognitive
load on prefrontal cortex of expert and novice surgeons. The authors
examined the potential predictive power of these fNIRS measurements for
determining the cognitive load as well as the expertise of their
participants in performing two laparoscopic surgery tasks: peg transfer
and threading.

Major Comments:

Data Analysis:**

**-- How did the authors compute the hemoglobin concentrations (e.g.,
beer-lambert, etc.)?**

OUR RESPONSE: The modified Beer-Lambert Law (MBLL) was applied to compute the concentration changes of HbO and HbR. The differential path length factor (DPF) values of 780 nm and 850nm are 5.075 and 4.64 in respectively. We provide an explanation of how we compute the haemoglobin concentrations in the updated preprocessing section. We have also cited the paper by Delpy 1988, Choi 2013 and Boas 2000.

-- **Also, the preprocessing of NIRS time series appear
to solely include bandpass filtering without any baseline normalization
and detrending (the latter for prevent potential non-stationarity in
time series).**

OUR RESPONSE: In our case the highpass filter (0.01 Hz) eliminated all baseline drift and any slow features (longer than 100 s). This was sufficient for the purposes of this study as our features were standard deviations computed within 10 s windows. The 10 second standard deviation was a mean subtracted measure hence not affected by any remaining slow components in the signal to our knowledge. The preprocessing stages and their justification are explained in greater detail in the new, expanded Data Analysis section.

**-- Another issue is with regards to the use of standard
deviation (3 in their case) for artefacts attenuation. This step is
quite unconventional and is not (to the best of reviewer's knowledge)
practiced in the literature. The authors are encouraged to consult [1]
for a comprehensive review of NIRS preprocessing.**

**[1]** **Tak, S. and Ye, J.C., 2014. Statistical analysis of fNIRS data: a
comprehensive review. Neuroimage, 85, pp.72-91.**

OUR RESPONSE: The standard deviation has in fact been used for artefact attenuation, for example Scholkmann 2010. In the next version of the manuscript we provide extensive description and justification of our method (together with a new figure). We have also cited the paper by Tak and Ye 2014.

**-- In addition, the authors also appear to use their measured NIRS standard
deviation (i.e., after they preprocessed it) for quantification of the
brain activation. This is also very unconventional. Specifically, what
the authors refer to as "PFC activation" throughout the manuscript is
indeed the deviation of the channels' activation from the average
(observed/induced) PFC activity. In fact, such deviation could still be
present without any sufficient/significant induced PFC activation by the
task. The authors are strongly encouraged to consult [2,3] for measures
used for NIRS quantification.

[2]** **Naseer, N. and Hong, K.S., 2015. fNIRS-based brain-computer
interfaces: a review. Frontiers in human neuroscience, 9, p.3.**

**[3] Keshmiri, S., Sumioka, H., Yamazaki, R. and Ishiguro, H., 2018.
Differential entropy preserves variational information of near-infrared
spectroscopy time series associated with working memory. Frontiers in
neuroinformatics, 12, p.33.**

OUR RESPONSE: The variance (closely related to the standard deviation used by our study) was described as one of the features available for use in fNIRS analysis by Naseer and Hong 2015. We have cited this paper in the new version of the manuscript.

Evoked hemodynamic response in a 10 s window this may be in the form of a rise in the oxyhemoglobin concentration followed by a dip. The within-window standard deviation is a sensitive measure for capturing this response. We provide an explanation of how in our study the standard deviation can be legitimately considered as a measure of PFC activation in the new subsection Feature extraction and selection

-- **With regard to the use of short-distance channels "to perform
superficial signal regression (SSR)" on long-distance channels, it is
not clear what methodology/approach the authors adapted in their study.
Some of the available approaches are:

[4] Fekete, T., Rubin, D., Carlson, J.M. and Mujica-Parodi, L.R., 2011.
The NIRS analysis package: noise reduction and statistical inference.
PloS one, 6(9), p.e24322.

[5] Zhang, Y., Brooks, D.H., Franceschini, M.A. and Boas, D.A., 2005.
Eigenvector-based spatial filtering for reduction of physiological
interference in diffuse optical imaging. Journal of biomedical optics,
10(1), p.011014.

[6] Kohno, S., Miyai, I., Seiyama, A., Oda, I., Ishikawa, A., Tsuneishi,
S., Amita, T. and Shimizu, K., 2007. Removal of the skin blood flow
artifact in functional near-infrared spectroscopic imaging data through
independent component analysis. Journal of biomedical optics, 12(6),
p.062111.

[7] Haeussinger, F.B., Dresler, T., Heinzel, S., Schecklmann, M.,
Fallgatter, A.J. and Ehlis, A.C., 2014. Reconstructing functional
near-infrared spectroscopy (fNIRS) signals impaired by extra-cranial
confounds: an easy-to-use filter method. NeuroImage, 95, pp.69-79.

[8] Gagnon, L., Perdue, K., Greve, D.N., Goldenholz, D., Kaskhedikar, G.
and Boas, D.A., 2011. Improved recovery of the hemodynamic response in
diffuse optical imaging using short optode separations and state-space
modeling. Neuroimage, 56(3), pp.1362-1371.

[9] Keshmiri, S., Sumioka, H., Okubo, M. and Ishiguro, H., 2019. An
Information-Theoretic Approach to Quantitative Analysis of the
Correspondence Between Skin Blood Flow and Functional Near-Infrared
Spectroscopy Measurement in Prefrontal Cortex Activity. Frontiers in
neuroscience, 13, p.79.**

OUR RESPONSE: In fact we used the method in Gagnon et al 2011. However due to an error in the manuscript the reference number showed another paper. This has now been fixed and we cite the Gagnon et al paper in the new subsection Feature extraction and selection.

**Analysis Steps with Machine Learning:

-- Although the authors mentioned the use of signal's standard deviation
for quantifying the brain activation, they then switched to its mean for
ML-based classification (page 8: "For this purpose feature matrices were
built with each row (an observation) representing the mean of the
prefrontal activation over an episode"). Such inconsistencies and mixing
of measures/metrics make quite difficult to realize the potential
underlying property of the signal based on which the results have been
derived.**

OUR RESPONSE: This in fact a misunderstanding due an unclear statement in the manuscript. The features we used by the standard deviations that were averaged over an experimental episode. Thus we did not switch to a new metric, but only averaged our metric over an episode. (The reason for doing so is related to another probing question raised by the reviewer, namely whether we included data from the same subject in both training and test sets. By averaging over an episode we insured that there was only one row per subject in the feature matrix.) We have changed the unclear statement in the subsection Classification by inserting the phrase “representing the standard deviations averaged over an episode”.

**-- The authors also mentioned that (page 9) "The feature types were
prioritised by using the Pearson correlation between the observations
and the labels, a standard feature-selection technique" - Do authors
refer to the channels as features? If so, this step actually decided on
which subset of channels to be used as inputs to their ML model.**

OUR RESPONSE: Yes, each channel contributes a separate column in the feature matrix.

**-- The authors continued by explaining that (page 9) "We then chose a small
group of features from the prioritised list and used it to train Support
Vector Machines (SVM) with linear kernels." - How did the authors decide
this "small group of features?" Did they apply such utilities as
"features importance" that are available through ML libraries? If so,
what criterion/criteria was/were used to determine the level of
significance of feature scores? (while using the term "feature," I am
assuming "selected channels" as per authors' earlier explanation).**

OUR RESPONSE: The Pearson correlation between a feature and the labels is a way of estimating the extent to which that feature will be helpful in classifying the labels. We have used this technique in previous studies e.g. Omurtag et al 2017 which provides references for it. But in the new version of the manuscript we provided additional details and referenced two other methods Minimum Redundance and Maximum Relevance and the Chi-square tests, available through Matlab. We explored these methods but they are more involved than the Pearson correlation without any clear benefits, hence were not adopted in our study.

**-- The authors also used 5-fold cross-validation for testing the accuracy
of their model. As the authors explained, every participant in their
study participated in two different tasks (i.e., Peg Transfer and
Threading). As such, did the authors ensure that data from the same
individual were not present in both train and test sets while applying
5-fold cross-validation? This is an important issue while performing
such analyses since data from the same individuals should not be
expected to be highly different between the two tasks which could, in
turn, results in overestimation of the model's accuracy.**

OUR RESPONSE: Thanks for pointing out this important issue. We ensured that the feature matrix contained only one row per subject for each classification problem. Therefore the training and test sets did not share data from the same subject. This is clarified in the new subsection Classification.

**Result:

-- As one of their hypotheses, the authors stated (page 9) "that there
would be differences in the activations due to the different sampling
depths of the channels with different separation distances." - The
effect of channel separation and skin- other than cortical-blood-flow is
a well-studied subject. In fact, short-distance channels are not
expected to represent cortical activity. Similarly, channels with
distances larger than 3.5 cm have been also generally accepted to not
produce reliable results due to the absorption of optical signals as it
penetrates deeper to cortical tissues. Please consult [5-9] above for
more in-depth results and discussion on this matter.**

OUR RESPONSE: We have added new statement and new references in the 3^rd^ paragraph of the Discussion section reminding the reader of degraded signal-to-noise associated with longer source-detector separations. The long separation we have used is 3.35 cm, which is well within the range of 25-35 mm which is considered typical in several fNIRS studies, which we provide references for. Although we realize that, as indicated by the reviewer, one should try to avoid the longer end of this range in order to avoid noise, as we have done.

**-- The authors' statement (page 10) "Figure 2 shows that in the Student
subjects who had experienced higher task load also had higher PFC
activations." that is quite repeated throughout the manuscript is not
really valid since the authors used the standard deviation of the
signal. In other words, these quantities are how individuals' PFC
activity deviated from the averaged observed/induced PFC activation in
their study.**

OUR RESPONSE: The reviewer correctly points out that the observed PFC activation is typically based on haemoglobin concentration changes. However we believe that for the purposes of this study, their standard deviation is an equally valid meaning of the term activation, because (with proper preprocessing) greater evoked hemodynamic responses resulted in greater standard deviations. In this version of the manuscript we have clarified this definition of “activation” in the beginning of the new subsection “Feature extraction and selection” in Methods with the sentence: “Because greater evoked hemodynamic response tends to increase the standard deviation of the signal in a window, we took these values as the measure of the prefrontal activation.” Because of this close relationship, introducing a different term (other than activation) might have been confusing to the readers. We respectfully would like to keep this term, as it has now been clearly defined in the new version of the manuscript; it does not dramatically differ from the typical interpretation, and it provides a concise and coherent way of describing our results.

**-- With regard to results' presentation, the authors sufficed to such
statements as (e.g., page 10) "The difference between high and low load
subjects were statistically significant in both Tasks in the case of the
shortest (1.5 cm, A and E) and the normal separation (3 cm, C and G)
channels." while referring to the figures 2 through 7 without providing
any descriptive statistics for their results. Precisely, it is not clear
how the authors determined these results "were statistically
significant?" What type of tests did they apply? What were the p-values,
test-statistics, mean, standard deviation, confidence intervals, and
effect-sizes associated with these tests? Did the authors corrected
their p-values while determining their significance (e.g., Bonferroni,
FDR, etc.)?**

OUR RESPONSE: Thanks for pointing out this gap in our manuscript. We have added new material under the new subsection Statistical analysis under the Methods section. We describe at length our approach there that let us determine the statistical significance of our results. In the new version we have also introduced Bonferroni correction to adjust the significance cut-off for the p-values. This new cut-off slightly affected the figures, hence we have introduced new versions of the box plot figures, where a few of the previously significant results appear without an asterisk. However the majority of the significance results still remain.

**--The authors stated that (page 11) "In addition, for the student subjects
there was a pronounced asymmetry in the case of the deepest sampling
channel (D and H), the activation on the left being significantly higher
than on the right." - The reviewer encourages the authors to consult the
studies related to the effect of short/long-distance channels on NIRS
measurements that are listed above. In particular, the "deepest sampling
channel" in the present study could fall within the range that is
considered not suitable for studying the cortical activation.**

OUR RESPONSE: In the 3^rd^ of paragraph of the Discussion section we address this issue and provide new references.

**-- With regard to the source localization presented in Figure 5, the
authors used such statements as (page 11) "there is a hint of high
activation localized near the top left …" - Such assertion are not
justified unless the authors provide statistical evidence for the
possibility of such activations that differ from the other regions.**

OUR RESPONSE: We thank the reviewer for pointing out this oversight in the manuscript. We have inserted in the new version a new paragraph immediately after Figure 5 which explains how to assess the statistical significance of Figure 5 based on the corresponding subplots of Figure 4. The greater activation in student subjects in the left PFC is significant by this analysis, as explained in the new paragraph.

**Above shortcomings with regard to the results' representation also apply
to the case of the results pertinent to ML-based results.

Discussion:

-- The authors stated that (page 13) "This difference, visible in most
channel separations, was statistically significant in the 1.5 cm and
most of the 3 cm separated channels (Figure 2)." - The authors did not
present sufficient statistics for this claim (only presenting figures).**

OUR RESPONSE: This gap in the manuscript has now been addressed by fully describing the statistical analysis in the new subsection Statistical analysis under the section Method, and by the new version of Figure 2 which includes clear indications of which group differences are significantly different.

**-- They also stated that (page 13) "in skilled subjects in the correlation
of PFC activation with subjective task" however, the reviewer could not
find/see these correlation analyses.**

OUR RESPONSE: To improve clarity this sentence has now been modified so that it reads: “This change in skilled subjects’ PFC response relative to that of unskilled ones may have been due to…”.

**-- Another issue is with regard to hemispheric differences that authors
referred to (page 14) "We found that response was greater in the left
PFC of students (Figure 4D and H), ..." - Unless the authors perform
statistical tests, such claims are not truly founded.**

OUR RESPONSE: We believe this valid critique has now been answered in the new version of the manuscript since we have a new section about the statistical analysis and we show the significant differences clearly on the figures. The difference mentioned in this comment is indeed statistically significant according to our analysis.

**Other Comments:

-- The language of the manuscript requires a thorough auditing and
proofread as it is not easy to follow and comprehend the study.**

OUR RESPONSE: We have introduced numerous clarifications and some stylistic edits (in addition to the corrections listed above).

**-- The quality of figures are very low and must be improved.**

OUR RESPONSE: The quality of figures are improved. We used PACE diagnostic tool to ensure that figures meet PLOS One requirements.

.

**-- Please also break your Results Section into different subsections (e.g.,
one for test of significant differences, another for ML-based results,
etc.) to help reader better follow and understand the results.**

OUR RESPONSE: We have introduced the new subsection titles “Group differences” and “Machine learning results” in the Results section.
