# Supplementary material for: High density optical neuroimaging predicts surgeons’s subjective experience and skill levels
Source: PLoS One. 2021 Feb 18;16(2):e0247117. doi: 10.1371/journal.pone.0247117 (PMC7891714; doi:10.1371/journal.pone.0247117)
Supplement: Attachment — Submitted filename: Response to Reviewers_Final.docx [file pone.0247117.s008.docx]

Dear Prof. Manabu Sakakibara,

**Thank you for sending the reviewer comments for our revised manuscript. We are very happy to see that they find the revised version greatly improved.**

**Regarding Reviewer 1:**

We used PACE diagnostic tool to ensure that figures meet PLOS One requirements. If there is a remaining problem with the figures, we will be happy to address them promptly. In the revised submission, figure captions were inserted in the text of manuscript. Because of PLOS One requirements, we did not include captions as part of the figure files.

**Regarding Reviewer 2:**

We appreciate this reviewer’s close scrutiny of our revised MS. The only remaining criticism from this reviewer is that the values in the results section should be given (in addition to the figures). We have now given the values as tables in Supp. Information section.

We believe the new version fully meets all reviewers’ requests; however we will be happy to address any remaining issues with the manuscript.

Sincerely,

Hasan Onur Keles, PhD
